# Gestational Diabetes Risk May Vary Depending on Birth Month

**DOI:** 10.3390/jcm14217756

**Published:** 2025-10-31

**Authors:** Eusebio Chiefari, Maria Mirabelli, Livia Cornelia Chiefari, Francesco S. Brunetti, Stefania Giuliano, Daniela P. Foti, Antonio Brunetti

**Affiliations:** 1 Department of Health Sciences, University “Magna Græcia” of Catanzaro, 88100 Catanzaro, Italy; echiefari@gmail.com (E.C.);; 2 Operative Unit of Endocrinology, “R. Dulbecco” University Hospital, 88100 Catanzaro, Italy; 3 Department of Medicine, University of Padova Medical School, 35129 Padova, Italy; 4 Department of Experimental and Clinical Medicine, University “Magna Græcia” of Catanzaro, 88100 Catanzaro, Italy; 5 Operative Unit of Clinical Pathology, “Renato Dulbecco” Hospital, 88100 Catanzaro, Italy

**Keywords:** gestational diabetes mellitus, month of birth, seasonality, Developmental Origins of Health and Disease (DOHaD) theory

## Abstract

**Background/Objectives**: Prenatal environmental exposure may influence disease risk later in life. Previous studies suggest that season or month of birth affects susceptibility to various conditions, including type 2 diabetes. We aimed to evaluate whether birth timing is associated with gestational diabetes mellitus (GDM). **Methods**: We conducted a retrospective cohort study of 8744 pregnant women screened for GDM between August 2011 and March 2020, according to Italian Ministry of Health guidelines. Only women born and raised in Calabria were included. Logistic regression and Cosinor analysis were performed. **Results**: Birth distribution peaked in January (30.7%) and was lowest in October (22.3%). Being born in January was associated with higher GDM [OR 1.287 (1.090–1.520), *p* = 0.003], whereas October and June births were protective [OR 0.800 (0.672–0.954), *p* = 0.013, and OR 0.818 (0.682–0.980), *p* = 0.030, respectively]. Birth in cold months increased GDM risk [OR 1.196 (1.080–1.325), *p* < 0.001], while birth in warm months was protective [OR 0.834 (0.758–1.917), *p* < 0.001]. Cosinor analysis of fasting glucose at OGTT confirmed significant seasonal periodicity (*p* = 0.0053). **Conclusions**: Season and month of birth are associated with GDM risk, cold-month births predisposing and warm-month births protecting. These findings suggest that early-life seasonal factors, potentially including maternal hyperglycemia during pregnancy, may influence future GDM risk.

## 1. Introduction

Gestational diabetes mellitus (GDM), defined as glucose intolerance first recognized during the second and third trimester of pregnancy [1], is the most common metabolic disorder in pregnancy, affecting more than 23 million pregnancies annually worldwide [2]. GDM is a major risk factor for maternal and neonatal complications and is associated with an increased long-term risk of type 2 diabetes mellitus (T2D) [3] and cardiovascular disease [4,5]. Women with a history of GDM have up to a seven-fold higher risk of developing T2D, and nearly one-third of women with T2D report a prior diagnosis of GDM [3].

Accumulating evidence suggests that GDM represents an early stage in a continuum of abnormal glucose regulation that may ultimately lead to T2D [6]. This view is supported by the observation that β-cell dysfunction in GDM is chronic rather than pregnancy-induced [6]. Although delivery usually leads to the resolution of hyperglycemia, more than 30% of women continue to display impaired glucose homeostasis in the early postpartum period [7]. Epidemiological and genetic studies further highlight the close relationship between GDM and T2D, showing shared genetic loci [8] and overlapping environmental risk factors [9].

Beyond genetic predisposition, prenatal environmental influences also play a role in the development of T2D in later life [10]. These include vitamin D synthesis through sun exposure, the nutritional quality of available foods, infectious exposure, and levels of outdoor physical activity [11,12,13,14,15,16,17]. Consistently, the risk of T2D has been linked to month of birth, reflecting seasonal variation in environmental exposures [18,19,20,21]. Although results are not always consistent [22], season of birth has been associated with several health outcomes, including life expectancy [23], final height [24], allergic diseases [25], schizophrenia [26], various cancers [27], obesity [28], and type 1 diabetes [29,30].

Despite these observations, only one study to date has examined whether month of birth influences the risk of developing GDM, and it found no association [31]. Therefore, the aim of the present study was to investigate whether the incidence of GDM varies by month of birth in a contemporary cohort of Mediterranean pregnant women from Calabria, Southern Italy. This population is of Caucasian ethnicity, is genetically homogeneous [32], and shows a higher prevalence of GDM, obesity, and T2D compared with the national average [33]. Our recent studies in this cohort demonstrated that the prevalence of GDM has doubled (from 13% to 28%) following the adoption of the International Association of the Diabetes and Pregnancy Study Groups (IADPSG) criteria [34,35,36].

## 2. Materials and Methods

### 2.1. Study Population

This monocentric, retrospective, population-based study included 8744 pregnant women who attended the tertiary care Endocrinology Unit of the “R. Dulbecco” Hospital, Catanzaro, Italy, between August 2011 and March 2020. To minimize selection bias, we included only women born and raised in Calabria, provided they were born from full-term pregnancies and that their mothers had spent the entire pregnancy in Calabria. Women who attended during the COVID-19 pandemic were excluded to avoid bias related to changes in screening practices.

Screening for GDM was performed using a 75 g oral glucose tolerance test (OGTT) at 16–18 weeks and/or 24–28 weeks of gestation, according to Italian guidelines [37]. These guidelines recommend selective screening based on maternal risk profile. Specifically, women at high risk (HR), defined as those with a history of GDM, pre-pregnancy body mass index (BMI) ≥ 30 kg/m^2^, or fasting plasma glucose (FPG) before pregnancy between 100 and 125 mg/dL (5.6–6.9 mmol/L)], underwent an early OGTT (16–18 weeks). Women at medium risk (MR), including those aged ≥35 years, with pre-pregnancy BMI of 25.0–29.9 kg/m^2^, with a history of macrosomia, with a first-degree family history of type 2 diabetes mellitus, or belonging to a high-risk ethnic group, as well as those with negative results for early OGTT, underwent OGTT later in pregnancy (24th–28th week). Women not meeting any of the above criteria were classified as low-risk (LR) and did not undergo screening. The diagnosis of GDM was established according to the IADPSG criteria: fasting glucose ≥ 92 mg/dL (5.1 mmol/L), 1 h ≥ 180 mg/dL (10.0 mmol/L), or 2 h ≥ 153 mg/dL (8.5 mmol/L) [38]. Gestational age was confirmed by ultrasonography examination.

Blood samples were processed within 30 min at the on-site clinical laboratory. Plasma glucose levels were measured using the enzymatic glucose oxidase method on the ILab650 chemistry analyzer (Instrumentation Laboratory, Werfen LLC, Bedford, MA, USA). The coefficient of variation was 1.5% at a mean glucose concentration of 73 mg/dL and 0.9% at 248 mg/dL, fulfilling recommended analytical precision standards.

The exclusion criteria were pre-existing diabetes mellitus, as defined by the American Diabetes Association (ADA) criteria, multifetal gestation, untreated endocrinopathies, active chronic systemic diseases, or use of medications known to affect glucose tolerance.

The study protocol was approved by the Ethics Committee of Regione Calabria.

### 2.2. Statistical Analysis

Quantitative variables were first tested for normality using the Shapiro–Wilk test. Continuous variables were compared between women with and without GDM, using the non-parametric Mann–Whitney U test, while categorical variables were compared using the two-tailed Fisher’s exact test. Spearman’s rank correlation was applied to assess associations between GDM and other parameters. All significant variables were included in multivariable regression models. Logistic regression analysis was used to evaluate the associations between GDM and seasonality, providing odds ratios (ORs) with 95% confidence intervals. Cosinor analysis was performed using an online tool (https://cosinor.online/appNew/index.php) (accessed on 12 August 2025). All other analyses were conducted with JASP Graphical Statistical Software, Version 0.17.2.0 (University of Amsterdam, Amsterdam, The Netherlands), based on R Stats packages.

## 3. Results

### 3.1. Characteristics of the Study Cohort

Table 1 summarizes the demographic, anthropometric, clinical, and biochemical characteristics of the enrolled women. Overall, 2275 of 8744 pregnant women (26.0%) were diagnosed with GDM: 240 (9.4%) at early screening and 2035 (90.6%) at later screening. Most affected women reported a family history of T2D in first-degree relatives, and a previous pregnancy. The proportion of non-Caucasian women was negligible (0.9%). Based on this stratification, 1517 (17.3%) were classified as HR, 5154 (58.9%) as MR, and 2073 (23.7%) as LR. Although our previous studies well support the importance of the early diagnosis and treatment of GDM in HR women [39,40], according to Italian guidelines, the proportion of HR women undergoing early screening was inappropriately low (33.3%). Among them, 240 (47.5%) received a GDM diagnosis at 16–18 weeks. Overall, GDM was diagnosed based on glycemia at time 0 during OGTT in 1444 women, at 60 min in 614 women, and at 120 min in 207 women.

### 3.2. Impact of Month of Birth on GDM Incidence

**Figure 1:**
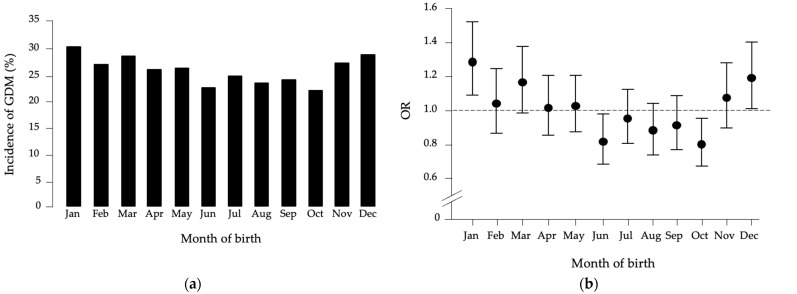
(**a**) Month-of-birth distribution of GDM in the studied population. (**b**) Odds ratio (OR) distribution with 95% confidence intervals for GDM patients versus healthy women, based on month of birth compared to all other months.

To investigate whether month of birth influences susceptibility to GDM, we compared GDM incidence across all pregnant women. As shown in Figure 1a, incidence was highest in January (30.7%) and lowest in October (22.3%). Logistic regression analysis, comparing each month with all others, revealed that being born in January was associated with increased GDM risk (OR = 1.287, 95% CI: 1.090–1.520, *p* = 0.003).

Conversely, being born in October or June was protective (October: OR = 0.800, 95% CI: 0.672–0.954, *p* = 0.013; June: OR = 0.818, 95% CI: 0.682–0.980, *p* = 0.030) (Figure 1b). No associations were found for other months. Adjustment for covariates, including pre-pregnancy BMI, maternal age, previous GDM, gravidity, polycystic ovary syndrome (PCOS), and gestational weight gain, did not alter these results.

To further substantiate these findings, we performed month-by-month comparisons. Significant differences emerged in age distribution (*p* = 0.042), in the proportion of MR women between November and February, and in the proportion of LR women born in April or November compared with those born in February and August (Table 2). No differences were observed in pre-pregnancy BMI, history of GDM, gravidity, PCOS, or gestational weight gain (Table 2).

Logistic regression analysis showed that the increased risk associated with January birth was significant compared with June, July, August, September, and October (Table 3). After adjustment for age and pre-pregnancy BMI, significance was also observed compared with April and May. Conversely, October birth was protective compared with November, December, January, March, and May, as well as February after adjustment for age. Similarly, June birth was protective compared with November, December, January, March, and May, this latter month after adjustment for age (Table 3).

### 3.3. Seasonal Pattern and Rhythmicity of GDM Risk

**Figure 2:**
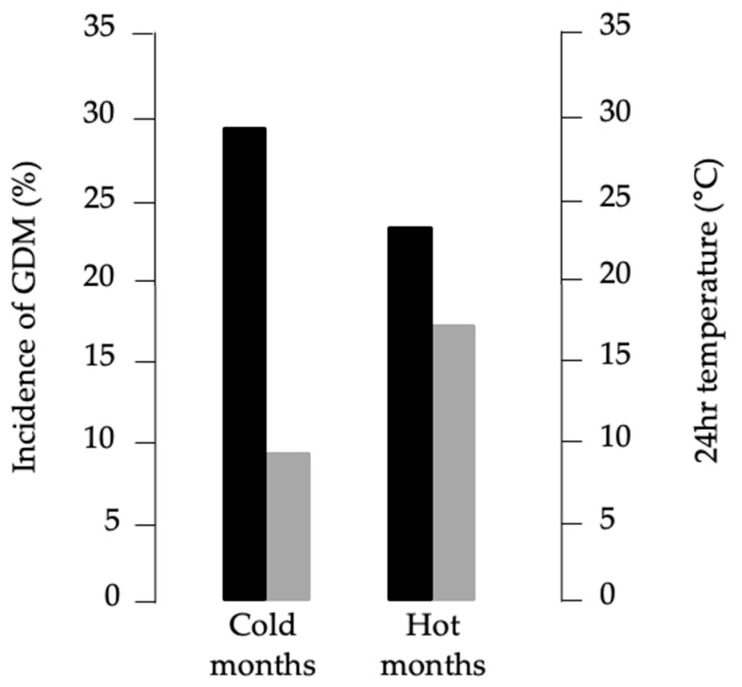
Relationship between GDM incidence and average monthly temperature. Black bars, percentages of GDM diagnosis; gray bars, 24 h average temperatures recorded at the local weather station.

These findings suggested a seasonal pattern of GDM risk. To test whether prenatal seasonal environment may influence future GDM susceptibility, we grouped women according to birth months characterized by average 24 h temperature ≥ 18 °C (May–October, “warm months”) or ≤12 °C (December–March, “cold months”), based on meteorological data from Catanzaro [41]. As shown in Figure 2, GDM incidence was higher among women born in cold months (28.8%) compared with warm months (24.2%, *p* < 0.001). Logistic regression confirmed that birth in cold months was a risk factor for GDM (OR = 1.196, 95% CI: 1.080–1.325, *p* < 0.001), whereas birth in warm months was protective (OR = 0.834, 95% CI: 0.758–1.917, *p* < 0.001). These results persisted after adjustment for pre-pregnancy BMI, maternal age, previous GDM, gravidity, PCOS, and gestational weight gain.

**Figure 3:**
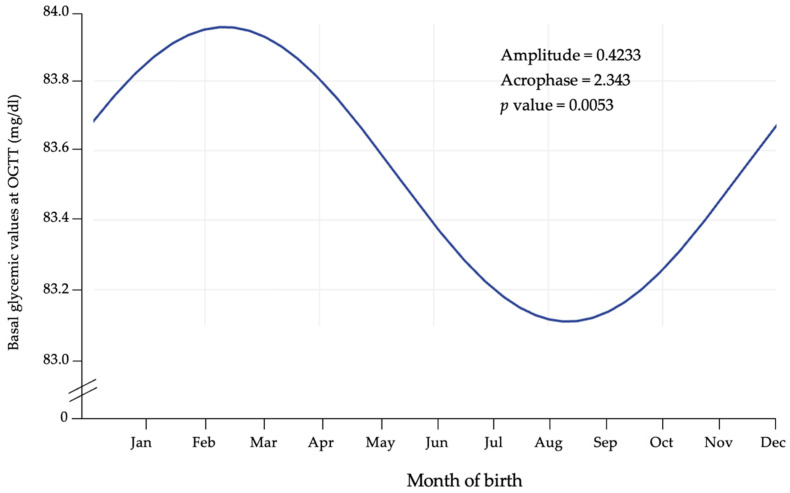
Cosinor fit plot of basal glycemia during OGTT.

To further validate this pattern, we applied Cosinor analysis to glycemic values measured during GDM screening at 0, 60, and 120 min. As shown in Figure 3, basal glycemic values displayed significant rhythmicity (*p* = 0.0053), with higher levels in cold months and lower levels in warm months, consistent with the incidence data. A similar but non-significant trend was observed at 60 and 120 min.

## 4. Discussion

In this study, we evaluated the association between month of birth and the risk of GDM in a Calabrian population. We found that women born in colder months, particularly January, had an increased risk of GDM, whereas those born in warmer months, especially in June and October, had a reduced risk. These associations were independent of maternal age, gravidity, previous GDM, or PCOS. Moreover, adjustment for baseline BMI and gestational weight gain did not materially alter the results, suggesting that adult adiposity does not mediate the effect of birth season on GDM risk, and that alternative mechanisms may be involved.

To our knowledge, only one previous study has investigated the relationship between season of birth and GDM, reporting no association. However, that study involved a population of different ethnicity, and used a distinct recruitment strategy. More evidence is available for T2D, which shares pathophysiological pathways with GDM. A Dutch hospital-based study of 282 T2D patients reported a higher incidence among individuals born in the first quarter of the year and a lower incidence among those born in the last quarter [18]. Similarly, a large Ukrainian study of 52,214 individuals found a peak in April and a nadir in November–December [19]. Prospective studies have yielded inconsistent findings. A Danish cohort of 223,099 adults reported no association between birth season and T2D [22], while a large Chinese cohort of nearly half a million participants demonstrated a lower risk among those born in summer [21]. The discrepancy may reflect differences in population feature characteristics, latitude, environmental exposure, and analytical adjustments. For instance, Denmark, at higher latitude, experiences fewer seasonal variations than China, which may partially explain the divergent results. Notably, our finding of a protective effect of warm-month birth aligns with the Chinese study, conducted at latitude more comparable to Calabria. Additional reports support similar trends, such as protective effects of October birth among African Americans [20] and a link between September–November birth and increased longevity [42].

Several mechanisms may explain our observations. Previous research on T2D has suggested that impaired fetal nutrition during late gestation may induce permanent changes in β-cell function or insulin sensitivity, predisposing to adult insulin resistance [43,44,45]. Although seasonal variations in food availability are not extreme in Calabria, differences in the nutritional value of seasonal foods and in eating habits across the three trimesters of pregnancy may determine epigenetic changes that influence the predisposition to future GDM [46]. Vitamin D exposure provides another plausible pathway. Sunlight, the primary determinant of vitamin D status, varies substantially across seasons. In Calabria’s temperate climate, sun exposure is particularly high from April to October. Consequently, women conceiving during spring or summer may attain higher vitamin D levels in early pregnancy, potentially conferring a protective effect against GDM in their daughters [47]. Currently, vitamin D supplementation is included in routine prenatal care. However, this practice has only recently been adopted and is not yet universal. Therefore, monitoring vitamin D levels in high-risk women, particularly during the colder months, may support earlier identification and mitigation of deficiency-related risks. Future studies should explore whether incorporating vitamin D-related parameters into risk stratification models improves predictive accuracy. Early postnatal factors may also play a role. Breastfeeding practices, which differ by season of birth [48], have been associated with long-term metabolic outcomes, including reduced risk of T2D [49].

An additional, intergenerational explanation deserves consideration. In our previous work, we reported higher GDM incidence in Calabria during warm months and lower incidence during cooler months [36]. Accordingly, mothers of women born in colder months would have undergone their critical gestational weeks (16–28) during summer, when undiagnosed maternal hyperglycemia may have been more prevalent, particularly before routine GDM screening was introduced in the 2000s. Such in utero exposure could predispose offspring to develop GDM later in life. This hypothesis is consistent with the Developmental Origins of Health and Disease (DOHaD) framework, which posits that early-life environmental factors can permanently shape metabolic risk [50]. Epigenetic mechanisms may mediate this process, as maternal hyperglycemia has been shown to alter DNA methylation in fetal cord blood [51,52]. Furthermore, meta-analytic evidence indicates that offspring exposed to maternal GDM have an elevated risk of metabolic syndrome in adulthood [53]. If confirmed, these findings highlight pregnancy as a dual opportunity for the prevention not only of T2D in mothers [54], but also of GDM in female offspring.

This study has some limitations. It is retrospective in design, and information on maternal health conditions, treatments, complications, and other exposures during pregnancy was unavailable. Nonetheless, several strengths should be emphasized: (1) the study population was genetically homogeneous and representative of the Calabria region; (2) GDM diagnosis was based on standardized IADPSG criteria; (3) all participants were born and raised in Calabria, limiting migration-related heterogeneity; (4) all OGTTs and biochemical analyses were performed in the same laboratory, ensuring consistency of diagnostic measurements.

## 5. Conclusions

In conclusion, our study demonstrates that birth month influences the risk of developing GDM, with a heightened risk among females born in colder months and a protective effect among those born in warmer months. Clinicians may consider incorporating seasonal factors and vitamin D status into comprehensive risk assessments, which could inform the timing of screening and preventive interventions. Further studies are necessary to confirm and extend these findings and to explore the underlying mechanisms linking season of birth to GDM risk in adulthood.

## Figures and Tables

**Table 1:** General characteristics of enrolled women.

Pregnant Women	N = 8744
Caucasian ethnicity (N)	8623 (99.1)
Age, yr	33 (29–36)
Age ≥ 35 (N)	3061 (35.0)
Family history of T2D (N)	3432 (39.2)
Pre-pregnancy BMI, kg/m^2^	24.0 (21.6–26.9)
Pre-pregnancy BMI ≥ 30 (N)	1021 (11.7)
Previous GDM (N)	575 (6.6)
Gravidity > 1 (N)	4578 (52.4)
PCOS (N)	563 (6.4)
HR women (N)	1517 (17.3)
MR women (N)	5154 (58.9)
LR women (N)	2073 (23.7)
Women who underwent early screening (N)	505 (5.8)
Women who underwent later screening (N)	8503 (97.2)
Women with GDM (N)	2275 (26.0)
GDM at early screening (N)	240 (10.5)
GDM at later screening (N)	2035 (89.5)
GDM diagnosis at time 0 during early OGTT (N)	206 (85.8)
GDM diagnosis at time 1 h during early OGTT (N)	25 (10.4)
GDM diagnosis at time 2 h during early OGTT (N)	9 (3.8)
GDM diagnosis at time 0 during later OGTT (N)	1238 (60.8)
GDM diagnosis at time 1 h during later OGTT (N)	589 (28.9)
GDM diagnosis at time 2 h during later OGTT (N)	198 (9.7)

Data are medians (IQRs) or N (%). BMI, body mass index; PCOS, polycystic ovary syndrome; OGTT, oral glucose tolerance test; HR, high-risk women; MR, medium-risk women; LR, low-risk women.

**Table 2:** Characteristics of enrolled women by month.

	N	GDM	Age	BMI	Obese	Overweight	PCOS	HR Women	MR Women	LR Women
January	720	221 (30.7)	32 (29–36)	23.6 (21.7–26.9)	77 (10.7)	202 (28.1)	50 (6.9)	136 (18.9)	420 (58.3)	164 (22.8)
February	636	170 (26.7)	32 (29–36)	24.0 (21.8–26.7)	73 (11.7)	173 (27.2)	44 (6.9)	109 (17.1)	361 (56.8)	166 (26.1)
March	737	212 (28.8)	32 (29–36)	24.0 (21.4–27.4)	87 (11.8)	223 (30.3)	60 (6.8)	122 (16.6)	431 (58.5)	184 (25.0)
April	735	193 (26.3)	33 (29–36)	24.1 (21.6–27.0)	95 (12.9)	212 (28.8)	50 (6.8)	137 (18.6)	440 (59.9)	158 (21.5) ^b^
May	834	221 (26.5)	33 (29–36)	24.0 (21.5–27.0)	102 (12.2)	232 (27.8)	53 (6.4)	146 (17.5)	493 (59.1)	195 (23.4)
June	721	163 (22.6)	32 (29–36)	24.1 (21.8–27.2)	91 (12.6)	197 (27.3)	45 (6.2)	123 (17.1)	417 (57.8)	181 (25.1)
July	803	202 (25.2)	33 (30–36)	24.0 (21.6–26.8)	91 (11.3)	241 (30.0)	52 (6.5)	147 (18.3)	472 (58.8)	184 (22.9)
August	770	183 (23.8)	32 (29–36)	24.0 (21.3–26.9)	88 (11.4)	218 (28.3)	48 (6.2)	123 (16.0)	447 (58.1)	200 (26.0)
September	733	179 (24.4)	33 (29–36)	23.8 (21.5–26.6)	81 (11.1)	203 (27.7)	48 (6.6)	122 (16.6)	445 (60.7)	166 (22.6)
October	785	175 (22.3)	32 (29–36)	24.0 (21.3–27.1)	100 (12.7)	206 (26.2)	47 (6.0)	137 (17.5)	453 (57.7)	195 (24.8)
November	667	182 (27.3)	33 (29–36)	23.9 (21.3–26.7)	68 (10.2)	185 (27.7)	42 (6.3)	106 (15.9)	418 (62.7) ^a^	143 (21.4) ^b^
December	603	174 (28.9)	33 (29–36)	24.5 (21.6–26.6)	70 (11.6)	172 (28.5)	40 (6.6)	109 (18.1)	357 (59.2)	137 (22.7)
*p* Value	-	-	0.042	0.790	-	-	-	-	-	-

The Kruskal–Wallis test was employed for comparisons of continuous variables. Fisher’s exact test was used for comparisons of categorical variables. ^a^, *p* < 0.05 after comparisons with February. ^b^, *p* < 0.05 after comparisons with February and August. BMI, body mass index; PCOS, polycystic ovary syndrome; HR, high-risk women; MR, medium-risk women; LR, low-risk women.

**Table 3:** Month-by-month comparison of association with month of birth.

	Jan	Feb	Mar	Apr	May	Jun	Jul	Aug	Sep	Oct	Nov	Dec
Jan	-	1.214 0.108	1.097 0.421	1.244 0.061 ^1^	1.228 0.068 ^1^	**1.516** **<0.001**	**1.318** **0.016**	**1.421** **0.003**	**1.371** **0.008**	**1.544** **<0.001**	1.396 0.163	1.092 0.467
Feb	0.824 0.108	-	0.704 0.401	1.024 0.844	1.012 0.921	1.249 0.079	1.085 0.498	1.170 0.202	0.978 0.328	1.272 0.053 ^2^	0.972 0.821	0.697 0.404
Mar	0.912 0.421	1.107 0.401	-	1.134 0.282	1.120 0.316	**1.382** **0.007**	1.201 0.111 ^2^	**1.295** **0.028**	1.250 0.060 ^2^	**1.408** **0.004**	1.076 0.538	0.996 0.971
Apr	0.804 0.061 ^1^	0.976 0.844	0.882 0.282	-	0.988 0.914	1.219 0.914	1.059 0.621	1.142 0.264	1.102 0.418	1.241 0.072	0.949 0.664	0.878 0.290
May	0.814 0.068 ^2^	0.988 0.921	0.892 0.316	1.012 0.914	-	1.234 0.076 ^2^	1.073 0.535	1.156 0.208	1.116 0.346	**1.259** **0.049**	0.961 0.732	0.889 0.323
Jun	**0.659** **<0.001**	0.800 0.079	**0.723** **0.007**	0.820 0.914	0.810 0.076 ^2^	-	0.869 0.245	0.937 0.596	0.904 0.415	1.018 0.884	**0.778** **0.044**	**0.720** **0.009**
Jul	**0.759** **0.016**	0.922 0.498	0.833 0.111 ^2^	0.944 0.621	0.931 0.535	1.151 0.245	-	1.078 0.552	1.040 0.739	1.172 0.180	0.896 0.355	0.829 0.121
Aug	**0.704** **0.003**	0.855 0.202	**0.772** **0.028**	0.876 0.264	0.865 0.208	1.067 0.596	0.928 0.552	-	0.965 0.767	1.087 0.490	0.831 0.127	**0.769** **0.033**
Sep	**0.729** **0.008**	1.022 0.328	0.800 0.060 ^2^	0.907 0.418	0.896 0.346	1.106 0.415	0.962 0.739	1.036 0.767	-	1.126 0.328	0.861 0.221	0.797 0.068
Oct	**0.648** **<0.001**	0.786 0.053 ^2^	**0.710** **0.004**	0.806 0.072	**0.794** **0.049**	0.982 0.884	0.853 0.180	0.920 0.490	0.888 0.328	-	**0.765** **0.028**	**0.707** **0.005**
Nov	0.716 0.163	1.029 0.821	0.929 0.538	1.054 0.664	1.041 0.732	**1.285** **0.044**	1.116 0.355	1.203 0.127	1.161 0.221	**1.307** **0.028**	-	0.925 0.534
Dec	0.916 0.467	1.435 0.404	1.004 0.971	1.139 0.290	1.125 0.323	**1.389** **0.009**	1.206 0.121	**1.300** **0.033**	1.255 0.068	**1.414** **0.005**	1.081 0.534	-

Odds ratios (upper) and *p* values (lower) after regression analysis are reported for each month. Significant results (*p* < 0.05) are represented in bold. ^1^, *p* < 0.05 after adjustment for age and pre-gravidic BMI. ^2^, *p* < 0.05 after adjustment for age.

## Data Availability

The original contributions presented in this study are included in the article. Further inquiries can be directed to the corresponding author.

## Abbreviations

The following abbreviations are used in this manuscript:

GDM	Gestational diabetes mellitus
IADPSG	International Association of the Diabetes and Pregnancy Study Groups
PCOS	Polycystic ovary syndrome
OGTT	Oral glucose tolerance test
BMI	Body mass index
HR	High-risk
MR	Medium-risk
LR	Low-risk
